# Unlocking the potential of RNA-based therapeutics in the lung: current status and future directions

**DOI:** 10.3389/fgene.2023.1281538

**Published:** 2023-11-23

**Authors:** H. S. Jeffrey Man, Vaneeza A. Moosa, Anand Singh, Licun Wu, John T. Granton, Stephen C. Juvet, Chuong D. Hoang, Marc de Perrot

**Affiliations:** ^1^ Temerty Faculty of Medicine, Institute of Medical Science, Toronto, ON, Canada; ^2^ Department of Immunology, University of Toronto, Toronto, ON, Canada; ^3^ Latner Thoracic Research Laboratories, Toronto General Hospital Research Institute, Toronto, ON, Canada; ^4^ Division of Respirology and Critical Care Medicine, Department of Medicine, University Health Network, Toronto, ON, Canada; ^5^ Division of Thoracic Surgery, Toronto General Hospital, Toronto, ON, Canada; ^6^ Thoracic Surgery Branch, National Cancer Institute, National Institutes of Health, Bethesda, MD, United States

**Keywords:** RNA-based therapy, mRNA vaccine, nanoparticle, noncoding RNA, antisense oligonucleotide, lung disease, RNA, COVID-19

## Abstract

Awareness of RNA-based therapies has increased after the widespread adoption of mRNA vaccines against SARS-CoV-2 during the COVID-19 pandemic. These mRNA vaccines had a significant impact on reducing lung disease and mortality. They highlighted the potential for rapid development of RNA-based therapies and advances in nanoparticle delivery systems. Along with the rapid advancement in RNA biology, including the description of noncoding RNAs as major products of the genome, this success presents an opportunity to highlight the potential of RNA as a therapeutic modality. Here, we review the expanding compendium of RNA-based therapies, their mechanisms of action and examples of application in the lung. The airways provide a convenient conduit for drug delivery to the lungs with decreased systemic exposure. This review will also describe other delivery methods, including local delivery to the pleura and delivery vehicles that can target the lung after systemic administration, each providing access options that are advantageous for a specific application. We present clinical trials of RNA-based therapy in lung disease and potential areas for future directions. This review aims to provide an overview that will bring together researchers and clinicians to advance this burgeoning field.

## 1 Introduction

The COVID-19 pandemic has publicized the value of RNA-based therapies because of the rapid development and widespread use of mRNA vaccines (Kojima et al., 2021). However, the potential of RNA-based therapy reaches far beyond mRNA vaccines, though the adoption of mRNA vaccines itself could be considered revolutionary. A major advantage of RNA-based therapies is that they drastically expand the numbers and types of targets that can be addressed therapeutically. While some proteins are considered difficult to target by other approaches, virtually all proteins and even noncoding RNAs are susceptible to RNA-based therapy (Bennett et al., 2017). RNA-based therapies can address genetic disease, with the ability to be highly specific, even targeting sequences with single-base pair mutations (Monga et al., 2017). Furthermore, RNA can be a vehicle for gene delivery into cells, whether as replacement therapy for protein-coding or noncoding genes or foreign sequences, such as with viral vaccines (DeWeerdt, 2019). The lungs are an attractive target for RNA-based therapy and can be accessed both by direct local delivery and systemic delivery. The goal of this review is to create a dialogue between lung-focused researchers, clinicians and developers of RNA-based therapy.

A major attraction of RNA-based therapies is that the primary nucleotide sequence forms the basis of the therapeutic effect. This principle has been best demonstrated by the development of COVID-19 mRNA vaccines created by Moderna and Acuitas/BioNTech/Pfizer, which took months rather than the years typically required for drug development (Kojima et al., 2021). Not only were the initial drugs developed quickly, but adaptation to new variants was fast based on the ability to generate a new “drug” once a different sequence was identified. This principle applies not only to mRNA vaccines but also to other mRNA therapeutics and to antisense therapies. Similarly, once delivery vehicles are developed that can target specific tissues and cell types, sequences for various targets can be rapidly developed to treat distinct disorders.

### 1.1 Timeline of development

Since early descriptions of RNA in the late 1950s (Rich and Davies, 1956), the field of RNA has grown to include many RNA classes, with functions ranging from encoding protein sequences to catalyzing enzymatic reactions and orchestrating gene regulation programs. Messenger RNA (mRNA) encoding for protein was described in 1961, a discovery which has anchored the central dogma of biology: that cellular information flows from DNA-to-RNA-to-protein (Crick, 1970). This perception of RNA biology dominated until the discovery of RNA interference (RNAi) and microRNAs (miRNAs) highlighted the critical role of small RNAs in gene regulation across species (Lee et al., 1993; Wightman et al., 1993; Fire et al., 1998).

The field of RNA therapeutics dates back as far as 1978 when Stephenson and Zamecnik designed an antisense oligonucleotide (ASO) that utilized RNA base-pairing to inhibit viral replication of the Rous sarcoma virus (Zamecnik and Stephenson, 1978). By 1990, the potential of mRNA transcripts as a vaccine was reported, after observing persistent gene expression in mice following *in vivo* injections of mRNA (Wolff et al., 1990). Further experimentation with mRNA led to the creation of a vaccine for the influenza virus (Martinon et al., 1993). In 1998, the FDA approved the first antisense RNA drug to treat cytomegalovirus retinitis (Roehr, 1998). Subsequently, in 2018, the first siRNA drug was approved for patients with hereditary transthyretin-mediated amyloidosis (Crooke et al., 2018). Despite these early successes, the potential of RNA-based therapy did not hit the public eye until 2020, when the first mRNA vaccines for COVID-19 were granted emergency use authorization, with full approval following in 2021 (Thompson et al., 2021). These therapies transformed the public health response to this rapidly emerging pandemic (Hogan and Pardi, 2022).

## 2 Spectrum of RNA-based payloads

**FIGURE 1:**
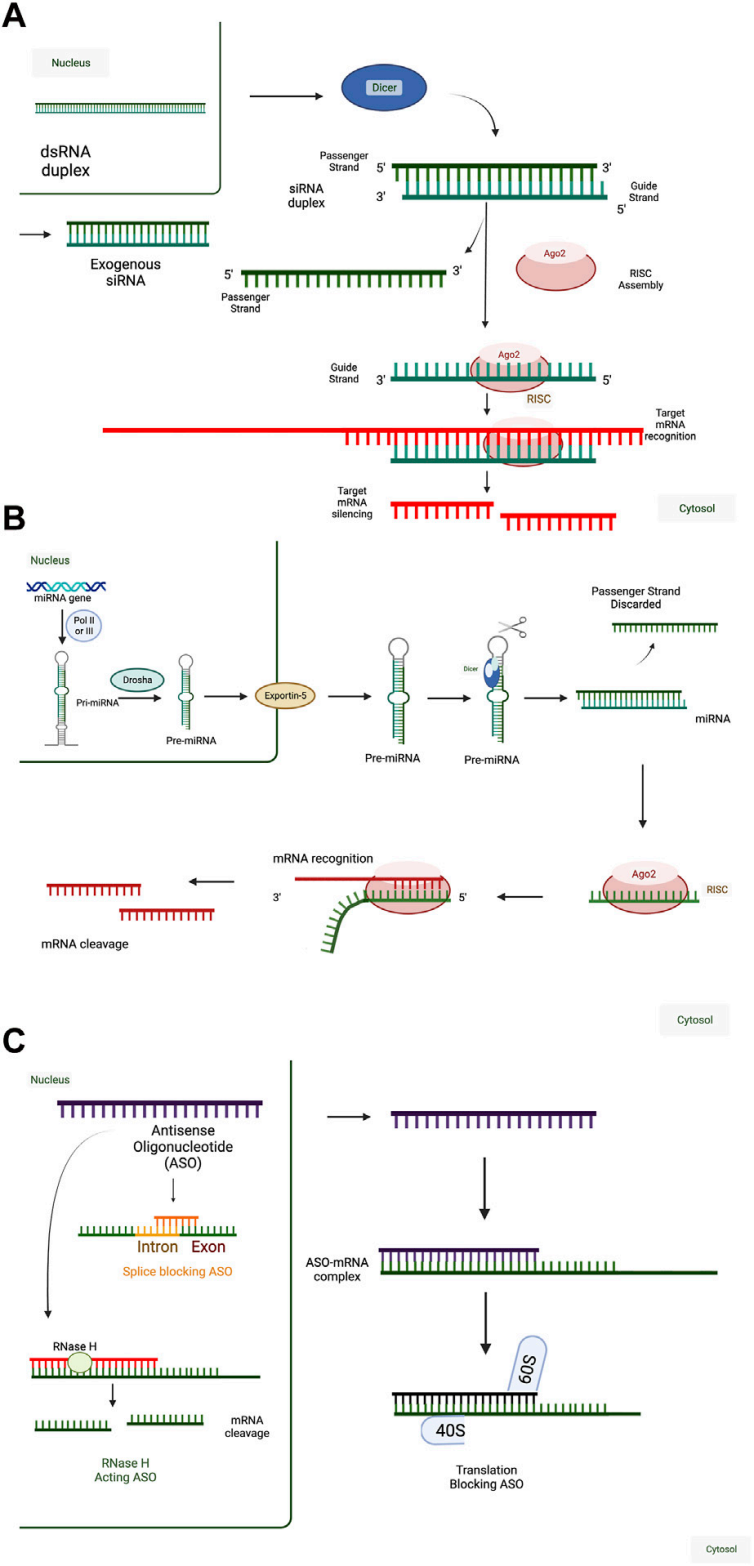
Mechanisms of RNA-Mediated Gene Regulation. **(A)** (Short-interfering RNA) siRNA mechanism of action. Double-stranded RNA (dsRNA) transcribed in the nucleus (e.g., from endogenous retroviral elements) are cleaved by Dicer in the cytoplasm into shorter RNA duplexes, which can interact with the RNA-induced silencing complex (RISC). Alternatively, exogenous siRNA (e.g., therapeutic siRNA) can interact with the RISC upon cellular entry. In both situations, the passenger strand of the siRNA duplex is discarded, and only the guide strand is incorporated in RISC. 100% complementarity of the siRNA sequence with the target mRNA leads to cleavage and degradation of the target mRNA. Therapeutic siRNAs are designed to act against one target mRNA and decrease target mRNA expression and function. **(B)** MicroRNA (miRNA) mechanism of action. In the canonical miRNA processing pathway, endogenous miRNAs are transcribed as primary miRNAs (pri-miRNA) and are processed by Drosha into shorter pre-miRNA sequences. Pre-miRNA sequences are then exported into the cytoplasm by Exportin-5 and processed into shorter, mature miRNA sequences by Dicer. Other miRNA processing pathways exist but are less common than the canonical pathway. Mature miRNA sequences, or exogenously administered miRNA duplexes, can interact with the RISC, discarding the passenger strand. miRNAs act through incomplete complementarity of the miRNA guide strand with target mRNAs and rely on a 7-8 nucleotide complementary “seed sequence,” typically in positions 2-8, to interact with a network of target mRNAs. Interaction of the miRNA-RISC complex with target mRNAs leads to translation inhibition or mRNA target degradation. Therefore, therapeutic miRNAs are designed to act against a network of target mRNAs and decrease target mRNA expression and function. **(C)** Antisense oligonucleotide (ASO) mechanism of action. ASOs are a diverse class of single-stranded, RNA-based therapeutics. ASO sequences can be formed from combinations of RNA, DNA, and modified nucleic acids in any order because their short sequences can synthesized in a base-by-base fashion (solid phase synthesis), as with miRNAs and siRNAs. Their mechanisms of action depend on both the chemistry, including base composition and sequence. ASOs can function in the nucleus or the cytoplasm and either decrease or increase target mRNA expression and function. ASOs with ∼10 nucleotide central DNA sequences flanked by ∼5 nucleotide RNA sequences with complete complementarity to the target mRNA can be used to decrease target mRNA expression by RNAse H. They can also be designed to bind intron-exon boundaries to block splicing to influence the expression of alternatively spliced RNA variants. In the cytoplasm, ASOs can be designed to block translation initiation sites of an mRNA. When these are designed against the primary translation initiation site, they will decrease the translation of the target mRNA into protein and when these are designed against upstream translation initiation sites that interfere with the primary translation initiation site, they will increase the translation of the target mRNA into protein. can impede translation by specifically targeting mRNA sequences, as translation blocking ASO. Created with BioRender.com.

**FIGURE 2:**
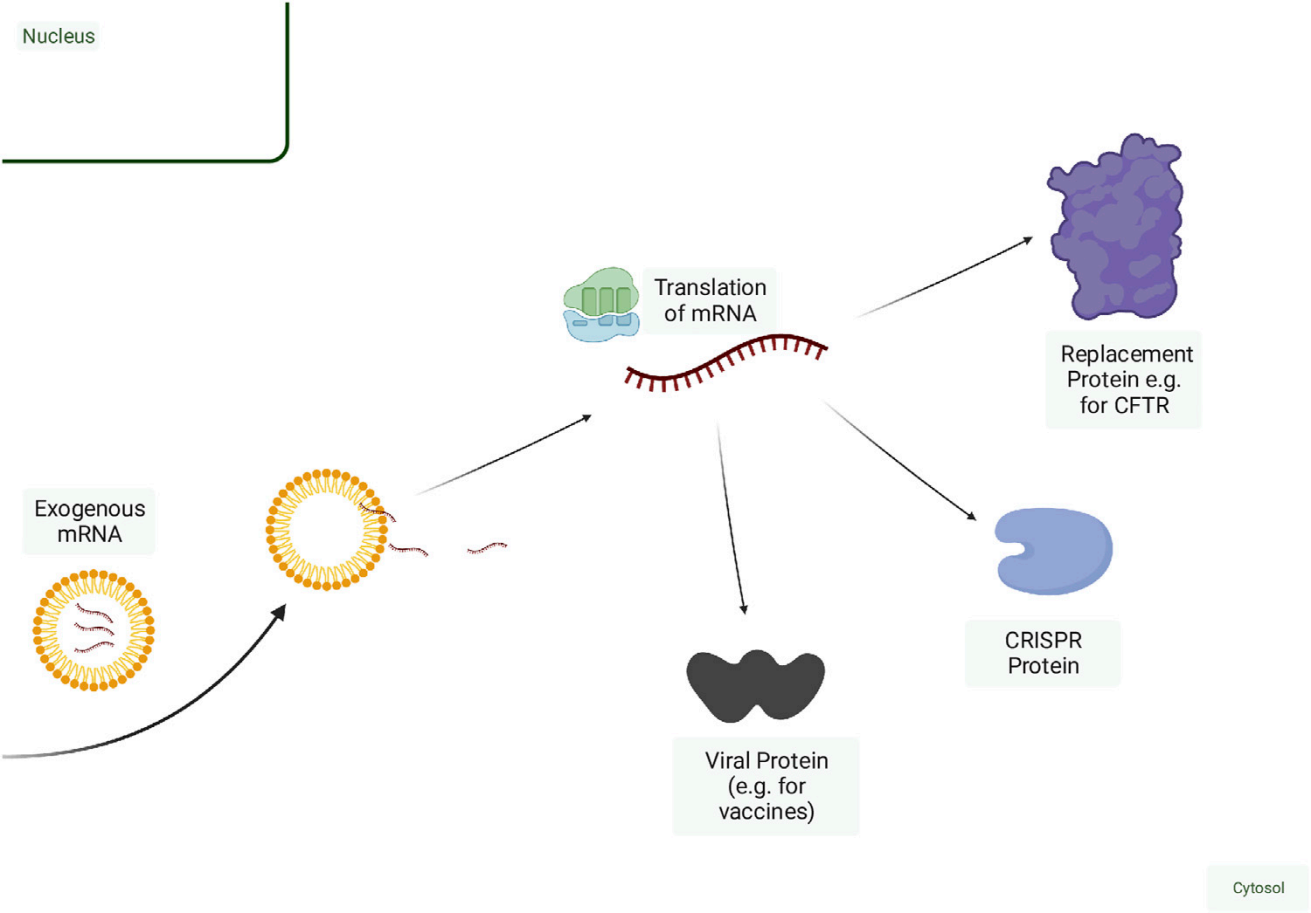
Mechanisms of mRNA-based therapy. Exogenous mRNA can be delivered naked but is more efficiently delivered within a delivery vehicle. Once released from the endosome, mRNA can then be translated into protein. Fragments of viral proteins or tumour antigens can be encoded in the mRNA for use as a vaccine. Deficient wild-type proteins can be replaced or augmented via mRNA delivery. mRNAs encoding CRISPR proteins along with a guide RNA can be delivered for genome editing or other adaptations of CRISPR function, including RNA degradation. Created with BioRender.com.

**FIGURE 3:**
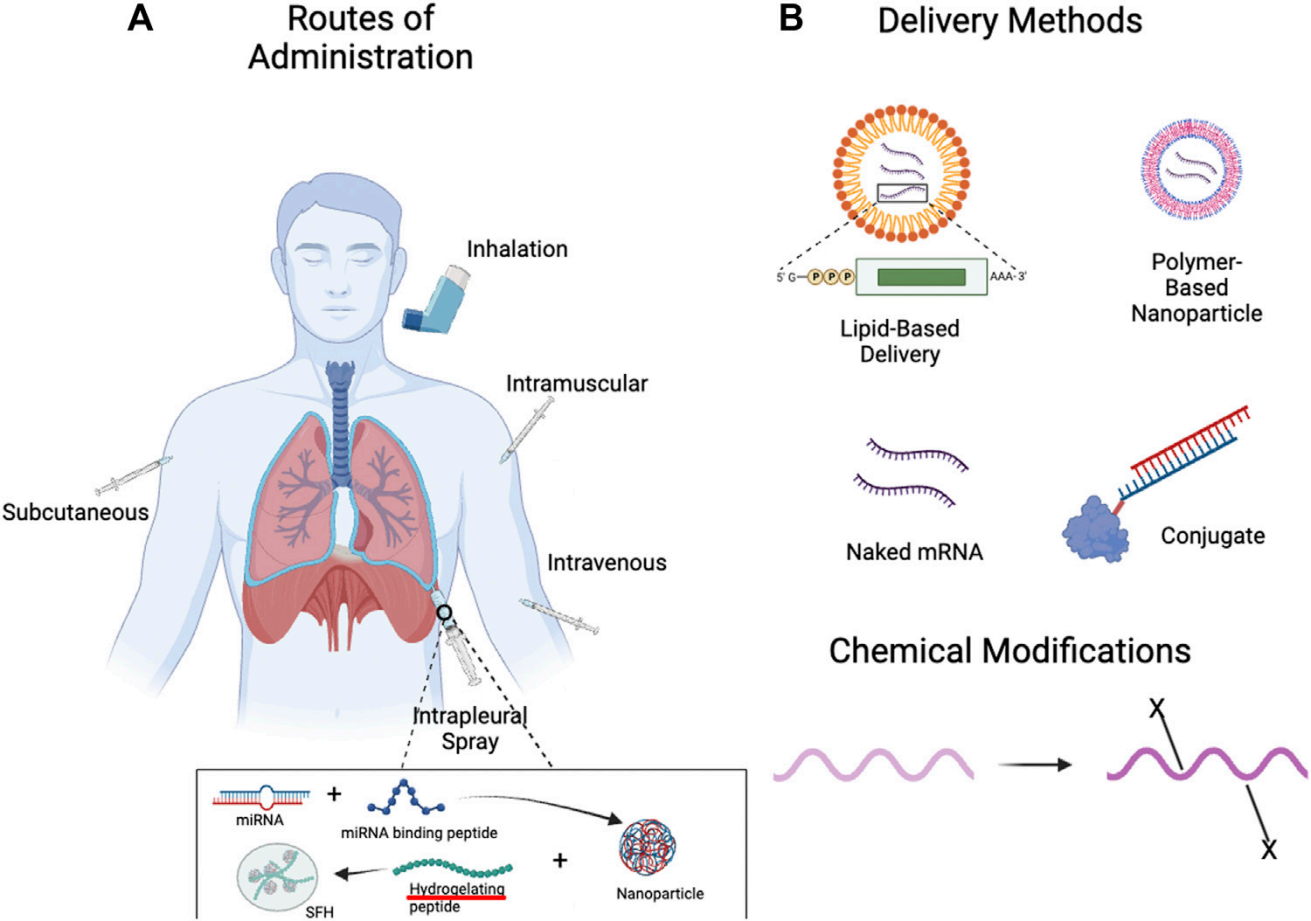
Methods of Administration and Delivery Methods for RNA-Based Therapies. **(A)** With advances in delivery vehicles, the lung can be targeted by RNA-based therapies through both local (inhalation, pleural spray/injection) (Majumder et al., 2021) and systemic delivery methods (intravenous, subcutaneous, *etc.*). **(B)** RNA-based therapies can be delivered naked, within delivery vehicles such as lipid- or polymer-based nanoparticles, and can also be conjugated with other moieties for the purposes of targeting. Chemical modifications can confer favourable properties, such as increased stability and reduced immunogenicity. Created with BioRender.com.

In theory, RNA payloads falling into any category of RNA (mRNA, microRNA, *etc.*) can be delivered therapeutically. In practice, current RNA-therapeutics fall into three broad categories: 1) RNA designed to inhibit target gene expression, often described as antisense RNA in reference to their mechanism of action via base-pairing (Figure 1); 2) RNA designed to express a protein, often referred to as mRNA-based therapy (Figure 2), and 3) those that target protein (RNA aptamers) (DeWeerdt, 2019). In this review, we focus on the first two categories. RNA aptamers are discussed elsewhere (Tuerk and Gold, 1990; Kang and Lee, 2013; Sundaram et al., 2013; Lei et al., 2023).

### 2.1 Antisense therapy: RNA-based therapy designed to inhibit gene expression

Most antisense therapies consist of short RNA molecules, often <30 nucleotides, termed oligonucleotides, and include short interfering RNAs (siRNAs), microRNAs (miRNAs), and antisense oligonucleotides (ASOs) (Crooke et al., 2018; Winkle et al., 2021). Antisense therapies target other nucleotides, typically other RNA species, through sequence complementarity following Watson-Crick base pairing rules (A:T/U, C:G) (Crooke et al., 2018; Winkle et al., 2021). Theoretically, this characteristic allows for targeting any known unique nucleotide sequence and positions antisense therapies well for developing treatments that target the ∼84% of proteins not currently druggable by other methods (Zhu et al., 2022). We will briefly review RNA therapies that act by: 1) degrading the target RNA through endogenous enzymes, such as the RNA interference (RNAi) pathway or RNAse H, or 2) mechanisms that do not involve degradation of the target RNA, such as modulation of RNA processing/splicing/polyadenylation and blocking translation into protein. While the double-stranded miRNAs and siRNAs are also “oligonucleotides” designed to act through Watson-Crick hybridization, the term ASO generally refers to single-stranded antisense oligos (Crooke et al., 2021).

Current antisense therapies typically consist of short nucleic acid molecules less than 30 nucleotides in length and can be produced through solid phase synthesis, which is performed in a base-by-base fashion (Juliano, 2016). Thus, the chemistry of nucleotides at specific positions can be adapted to confer desired properties, providing greater versatility and precision in the design of these treatments.

#### 2.1.1 RNAi

RNAi leverages the endogenous RNAi pathway by which mRNA cleavage and degradation is initiated by the RNA-induced silencing complex (RISC) (Lingel and Izaurralde, 2004). Endogenous RNA species that utilize the RNAi pathway include microRNAs (miRNAs), short-interfering RNAs (siRNAs), or Piwi-interacting RNA (piRNA), all of which are short non-coding RNAs (ncRNAs) that are involved in post-transcriptional gene silencing rather than encoding proteins (Fire et al., 1998; Bartel, 2004; Girard et al., 2006). Mature miRNAs and siRNAs are double-stranded ncRNAs, measuring 20–25 base pairs in length. They are loaded onto the RNA-induced silencing complex (RISC) and lead to mRNA degradation and/or inhibit translation, thereby inhibiting the expression of specific genes (Watts et al., 2008; Ferguson et al., 2020; Tian et al., 2021) (Figure 1). This approach of targeting and degrading specific mRNA sequences can be used to control gene expression and provides a promising method for treating a wide range of diseases.

##### 2.1.1.1 miRNA

miRNAs are endogenous small, non-coding RNA molecules that play a crucial role in regulating gene expression. There are currently thought to be 2,656 human miRNAs (miRDB - Statistics, n.d.) (Chen and Wang, 2020) predicted to target 29,161 unique genes. miRNAs are usually transcribed as longer, primary miRNA sequences before being processed into mature double-stranded miRNA that can interact with RISC (Figure 1) (Shukla et al., 2011). One strand is often favoured for incorporation into the RISC complex and silence targets with complementarity to that strand. The RISC contains one strand of the miRNA and various proteins such as Dicer, TRBP, PACT, and Argonaute (Bartel, 2004; Carthew and Sontheimer, 2009). The complex binds to specific sequences on target mRNAs based on a 7-8 nucleotide seed sequence (Doench and Sharp, 2004) on the miRNA and regulates the expression of target mRNAs by translational repression or mRNA degradation [or deadenylation] (Figure 1)]. Because of this short seed sequence, miRNAs typically target multiple genes, and therapeutic applications involving miRNAs can be an efficient strategy to modulate the expression of a set of genes within a miRNA regulatory network (Bartel, 2009; Bartel, 2018).

Although the seed sequence is central to miRNA function, other factors also influence which mRNA targets are regulated. Many miRNAs are expressed in a highly tissue-specific fashion and contribute to tissue-specific expression patterns (Sood et al., 2006). There is also cell- and context-specific action of miRNAs, whereby binding of miRNA to targets is variable across cell types and timelines (Ho et al., 2013; Nowakowski et al., 2018). In this context, the targeting of mRNAs can depend on the level of expression of the mRNA vs. miRNA and RNA-binding proteins that can occupy miRNA binding sites (Ho et al., 2013; Ho et al., 2021). miRNA families consist of multiple miRNAs which share a seed sequence but can have overlapping and distinct target genes and subcellular localization (Horita et al., 2021). These factors are important considerations for the design of miRNA-based therapies.

Several types of RNA-based therapy can leverage the biology of miRNAs and can either potentiate or inhibit miRNA function. miRNA mimics are synthetic miRNAs that can be used to repress a set of miRNA-regulated genes, whereas antisense oligonucleotides such as antagomiRs or blockmiRs are synthetic oligonucleotides that bind to miRNAs or compete for their target sites on mRNAs respectively (Krützfeldt et al., 2005; Davis et al., 2007). miRNA sponges contain multiple miRNA binding sites and can be used to sequester miRNAs from target genes (Ebert et al., 2007)).

miRNA-based therapy has been assessed in non-malignant and malignant lung disease in preclinical studies. miRNAs in the miR-29 family are downregulated in pulmonary fibrosis (Cushing et al., 2015). MRG-229 is a miR-29 mimic that decreases the expression of pro-fibrotic genes in animal models of pulmonary fibrosis (Chioccioli et al., 2022). The multiplexing potential of RNA-based therapies and delivery systems has been leveraged in models of non-small cell lung cancer (NSCLC). To simultaneously target mutation in the Kirsten ras sarcoma viral oncogene homolog (KRAS) and loss of p53 in a single-nanoparticle platform, miR-34a, siKras and cisplatin were formulated layer-by-layer into a multifunctional nanoparticle. This multifunctional nanoparticle therapy was preferentially distributed to the lung and prolonged the survival of mice in an orthotopic lung cancer model (Gu et al., 2017). These early studies demonstrate the versatility and specificity of miRNA-based therapeutics in lung disease.

##### 2.1.1.2 siRNA

siRNAs are a class of double-stranded RNA molecules that can silence specific genes by binding to complementary sequences in the mRNA and triggering degradation or inhibition of translation. Endogenous siRNAs exist and differ from miRNAs in that they are not derived from primary miRNA transcripts, and they do not undergo canonical miRNA processing. Rather, they can be derived from long double-stranded RNA (dsRNA) which have a variety of sources and are cleaved by Dicer to form siRNAs (Carthew and Sontheimer, 2009). In this context, endogenous RNAi acts as a natural defence against viral infections and transposable elements (Boudreau et al., 2011).

siRNAs are also derived exogenously and are used for gene knockdown in research and RNA-based therapeutics. In contrast to miRNAs, siRNAs typically have 100% sequence complementarity with their target genes (Lam et al., 2015). As a result, appropriately designed siRNAs theoretically have a single target, whereas miRNAs have multiple targets. Aside from selectivity for a single target, this feature can be applied to allele-specific gene silencing, where a pathogenic gain-of-function mutation on one allele can be targeted while sparing the wild-type allele, even with a single nucleotide difference in sequence (Monga et al., 2017).

Despite this theoretical single-sequence specificity, siRNA can still have off-target effects (Woolf and Costigan, 1999). One proposed mechanism is that siRNA sequences can function with incomplete complementarity, with 6-7 nucleotide sequences within the siRNA effectively acting as seed sequences, analogous to miRNAs (Birmingham et al., 2006). Such short seed sequences can target multiple genes. These effects can be minimized by choosing sequences predicted to have fewer off-target effects and by appropriate control experiments, including the assessment of mulitple siRNA sequences for a single target. Chemical modifications can also reduce off-target effects from seed sequence matching (Jackson et al., 2006). Other off-target effects from immune activation and saturation of the RNAi machinery are discussed in more detail later.

siRNAs are a powerful tool in the knockdown of target RNAs and have been a frequently used tool in the research setting to assess gene function in a variety of biological systems (Mello and Conte, 2004). Like miRNAs, siRNAs act through the RISC complex (Carthew and Sontheimer, 2009). One aspect of siRNA design is that the guide strand of the siRNA cannot be chemically modified as it interferes with its incorporation into the RISC. On the other hand, the passenger strand of the siRNA can be chemically modified to ensure its stability (Kuijper et al., 2021).

#### 2.1.2 Antisense oligonucleotide (ASO)

ASOs are a more chemically and mechanistically diverse group of molecules compared to miRNAs and siRNAs, both of which act through the RNAi pathway. ASOs are single-stranded molecules that can alter mRNA expression through a variety of mechanisms, including ribonuclease H (RNAse H) mediated decay, or steric hindrance of splice-sites or translation initiation (Bennett and Swayze, 2010; Crooke et al., 2017). Depending on the design and thus the mechanism of action, ASOs can either downregulate target gene expression through RNAse H or translation inhibition or upregulate target gene expression by increasing translation efficiency or by modulation of splicing (Rinaldi and Wood, 2018; Kim N. et al., 2020). Like siRNAs, ASOs can also be made through solid-phase synthesis.

The mechanism of action can be modulated by ASO biochemistry is a determinant of the mechanism of action. RNAs H is an endogenous enzyme that cleaves the RNA strand in an RNA-DNA duplex (Raal et al., 2010; Roshmi and Yokota, 2019). Thus, RNAse H-dependent ASOs are formed of or contain DNA sequences to target an mRNA. Off-target RNA cleavage can occur due to partial complementarity of only 6-7 nucleotides. Thus, a 20-nucleotide ASO formed completely of deoxynucleic acid (DNA) nucleotides can lead to cleavage of off-target mRNAs (Di Fusco et al., 2019). Thus, to reduce off-target effects, chimeric structures known as gapmers have been developed, with a central 10-nucleotide region of DNA flanked by regions of five RNA-like nucleotides that will not activate RNAse H (Bennett et al., 2017).

ASOs that act as steric blockers in an RNAse H-independent fashion (Figure 1) consist of RNA or modified RNA-like bases without a region of DNA (Bennett et al., 2017; Bandyopadhyay et al., 2018; Chandra Ghosh et al., 2018). Often, they utilize modified chains of synthetic nucleic acids intended to achieve greater stability and longer half-lives (Østergaard et al., 2015), and some have the potential to be delivered without a delivery vehicle (Bennett and Swayze, 2010). These non-enzymatic mechanisms include splicing modulation or inhibition of translation and require that ASO sequences target splice sites and start codons of translation, respectively.

ASOs that bind to intron-exon junctions in pre-mRNA destabilize splicing sites or displace/recruit splicing factors, and result in the exclusion or inclusion of certain exons (Bandyopadhyay et al., 2018; Chandra Ghosh et al., 2018). Open reading frames (ORFs) in the 5′-untranslated region (5′-UTR) of mRNAs, known as uORFs, can affect the translation efficiency of the primary open reading frame (ORF) into protein (Wang et al., 1999), and exist in over half of all human mRNAs (Calvo et al., 2009; Bottorff et al., 2022). ASOs can increase the expression of proteins encoded by target mRNAs by blocking the activity of uORFs, thereby increasing translation efficiency (Liang et al., 2016).

The FDA has approved several ASOs outside of lung disease, such as Nusinersen and Mipomersen (Kim, 2022). In non-malignant lung disease, ASOs have been used to target transforming growth factor-beta (TGF-β) (Kim J. et al., 2020) and NLRP3 (Bai et al., 2019), with the latter having the potential for prevention but not reversal of pulmonary fibrosis. ASOs have also been used to target Jagged 1, a protein involved in the development of goblet cell metaplasia in the lungs, showing decreased mucus production and reduced goblet cells in mice (Carrer et al., 2020). In SARS-CoV-2, ASOs that target a conserved region among immune evasive variants can inhibit viral replication and increase the survival of transgenic mice with human ACE2 (Vora et al., 2022). Other studies have also identified conserved sites in SARS-CoV-2 variants amenable to targeting by ASOs (de Jesus et al., 2021).

### 2.2 mRNA

The most widespread applications of mRNA therapeutics are the mRNA-based vaccines against SARS-CoV2 (Paunovska et al., 2022). mRNAs are typically longer RNA molecules, with eukaryotic mRNAs being ∼2,900 nucleotides on average (Guttman et al., 2010). mRNA therapeutics can encode parts of genes, as with vaccines or whole genes, and are used to introduce genetic information into cells (Rohner et al., 2022) (Figure 2). Because they are not produced on a base-by-base basis via solid-phase synthesis, there are limitations to generating mRNAs with site-specific chemical modifications (Paunovska et al., 2022). However, the introduction of chemically modified nucleotides can improve stability and reduce immunogenicity. For example, twice weekly injection of surfactant protein B (SP-B) mRNA with only 25% replacement of uridine and cytidine with 2-thiouridine and 5-methyl-cytidine can restore surfactant expression to 71% of wild-type in SP-B deficient mice (Kormann et al., 2011). Self-amplifying RNA (sa-RNA) vaccines are a form of mRNA-based vaccines (Frederickson and Herzog, 2021) that encode an RNA-dependent RNA polymerase; this allows for self-replication of the transcript, leading to higher levels and longer duration of protein expression.

When introducing genetic information, such as in vaccines or CRISPR-Cas9-based approaches (Gillmore et al., 2021), an advantage of mRNA therapies compared to DNA-based therapeutics is the short duration of protein production due to the shorter half-life of mRNAs compared to DNA vectors (Pardi et al., 2015), and lower risk of incorporation into the genome (Schlake et al., 2012). In applications that involve DNA editing (e.g., with CRISPR-Cas9), long-term expression from DNA payloads may lead to more off-target editing events, and there is the potential for DNA to integrate into the genome (Hanlon et al., 2019; Chen et al., 2020). In the lung, non-vaccine mRNA therapies are under development. One example is the development of inhaled mRNA to enhance cystic fibrosis transmembrane receptor (CFTR) expression in cystic fibrosis (Damase et al., 2021; mRNA Technology: Vaccines and Beyond - Sanofi, n.d.).

Beyond vaccines for infectious diseases, mRNA vaccines can be designed as cancer vaccines. For example, dendritic cells can be loaded *ex vivo* with mRNA coding for tumour-associated antigens and administered back to patients to elicit an immune response (Schumacher and Schreiber, 2015; Sahin et al., 2017).

### 2.3 Other RNA payloads

Other RNA classes and RNA-based molecules have been assessed for therapeutic potential. For example, circular RNAs (circRNAs) are a group of noncoding RNAs that have covalently joined ends and are present in all studied eukaryotic organisms (Sanger et al., 1976; Wilusz, 2018; Pfafenrot et al., 2021). Another examples stems from the discovery that RNA moieties formed the catalytic subunit of ribonuclease P provided evidence that nucleic acids can have inherent enzymatic activity (Kruger et al., 1982; Guerrier-Takada et al., 1983; Guerrier-Takada and Altman, 1984). Since then, at least 21 ribozyme families have been identified, including Hovlinc (human protein vlincRNA localization), a recently evolved class of ribozymes found in human ultra-long intergenic non-coding RNA (Chen et al., 2021; Deng et al., 2023). These discoveries have sparked interest in designing RNA-cleaving nucleic acid therapeutics as gene-silencing agents with modified nucleic acid bases conferring improved biological activity. However, challenges still exist in this field (Wang, 2021).

As with vaccines, genetic information traditionally delivered via DNA-based molecules can be delivered via RNA-based therapies. CRISPR-Cas systems were first identified as prokaryotic adaptive immunity that protects against phages and has been widely adopted in research for genome editing (Doudna and Charpentier, 2014). CRISPR-Cas systems adapted for application in eukaryotic systems are composed of a targeting RNA (guide RNA, sgRNA) and a Cas enzyme. The sgRNA, together with the Cas enzyme, can be delivered as DNA via a viral vector or as RNA via a lipid nanoparticle. Some Cas enzymes target RNA, including long noncoding RNAs, rather than DNA and result in RNA knockdown without relying on endogenous RNAi machinery (Konermann et al., 2018; Li G et al., 2021).

### 2.4 Challenges and solutions

#### 2.4.1 Activation of immune response

Double-stranded RNA (dsRNA) is a potent activator of innate immune responses that form a natural defence against viral RNA and transposable elements (Sadeq et al., 2021). Thus, the introduction of foreign dsRNA species can/will induce an immune response. Indeed, siRNA-based RNA therapeutics have commonly been seen to induce an innate immune response (Marques and Williams, 2005; Sioud, 2007; Robbins et al., 2008; Morral and Witting, 2012; Meng and Lu, 2017); this unintended effect can confound experimental results and contribute to side effects in a pharmacologic context (Olejniczak et al., 2011). siRNA structure, sequence, and the method of delivery can all contribute to an immune response. In particular, GU-rich sequences similar to viral RNAs are described to activate RNAi in cells (Meng et al., 2013).

Several strategies have been employed to mitigate the issues that can arise and lead to unintended immune stimulation. One is to avoid using sequences with known immunostimulatory motifs, for example, 5′-UGU-3′, 5′-UGUGU-3′ (Judge et al., 2005), and 5′-GUCCUUCAA-3′ (Hornung et al., 2005). Another method seen is to design siRNAs with modified nucleotides that reduce unwanted immune activation; incorporating certain modified nucleotides into the siRNA, such as 2′-O-methyl purines, 2′-fluoropyrimidines, and terminal inverted-dT bases can prevent immune activation (Morrissey et al., 2005).

#### 2.4.2 Saturation of the endogenous RNAi machinery

The endogenous RNAi machinery is critical for normal gene regulation by the miRNAs present in a cell at any given time. When exogenous siRNAs or miRNAs are introduced for research or therapeutic applications, there is the potential to saturate a significant proportion of the endogenous RNAi machinery (i.e., RISC) and competitively inhibit the function of endogenous miRNAs. In this way, exogenous administration of siRNA or miRNA can lead to the upregulation of endogenous miRNA target mRNAs (Khan et al., 2009). Saturation of this critical cellular machinery can have significant consequences *in vivo* (Grimm et al., 2006). Therefore, methods to assess the effects of different doses of RNAs on direct and indirect gene regulation to avoid RISC saturation can be employed (Grimm et al., 2006).

#### 2.4.3 RNA stability

RNA is susceptible to degradation by nucleases (Chioccioli et al., 2022). Specific chemical modifications can be made to the backbone to increase RNA stability. For instance, by substituting sulphur atoms for one of the non-bridging oxygen atoms in the internucleotide phosphate groups or through 2′-O-methyl or 2′-Fluoro-ribose sugar modifications, the resistance to nuclease degradation is enhanced and the circulation time is prolonged (Gaus et al., 2019). Bioactive molecules, such as cholesterol and lipids, can also be covalently attached to oligonucleotides in order to increase directed delivery (Hammond et al., 2021). For example, cholesterol conjugates increase delivery to the liver while reducing delivery to the kidney (Bennett et al., 2017).

## 3 Vehicles for RNA delivery

The potential versatility of RNA therapies presents great opportunities for disease treatment but requires that the RNA payload is delivered to the appropriate cells. Because of their negative charge, RNA molecules will encounter barriers in crossing cell membranes, and native RNA is susceptible to the ubiquitous ribonucleases (RNases) present in the body (Chioccioli et al., 2022). Chemical modifications can improve RNA stability but are generally applicable only to short RNAs that can be synthesized by solid phase synthesis (Gaus et al., 2019). Therefore, small oligonucleotides may be chemically modified to increase stability in the absence of an external vehicle whereas mRNAs must be delivered within a vehicle (Paunovska et al., 2022). Compared to DNA-based carriers of genetic information, which require transcription to RNA in the nucleus, many RNA-based approaches have the advantage that they can be delivered to the cytoplasm to exert their function without the need for nuclear delivery (Schott et al., 2016). As such, viral vectors such as adeno-associated virus (AAV) can be efficient delivery vehicles for RNA but have the disadvantage of triggering adaptive immunity (Tomar et al., 2003).

The development of nanotechnology-based delivery strategies is a critical component of effective RNA-based treatments (Figure 3). Nanotechnology is defined by structures roughly 1–100 nm in at least one dimension, though the term often refers to structures up to several hundred nanometers in size (Farokhzad and Langer, 2009). Delivery vehicles are designed to protect RNA from degradation, deliver the RNA payload to the appropriate cell type, and allow uptake of the RNA payload into the cell via endocytosis while minimizing toxicity (Cheng et al., 2015). Nanotechnology products can also co-deliver multiple payloads and be visualized at delivery sites with imaging (Farokhzad and Langer, 2009). Common nanotechnology vehicles include lipid-based nanoparticles (LNPs), polymer-based nanoparticles and peptide-based nanoparticles. A comprehensive discussion of delivery vehicles is out of the scope of this article and reviewed elsewhere (Paunovska et al., 2022). Here, we will present a selection of delivery vehicles and, where applicable, highlight target specificity relevant to the lung.

### 3.1 Lipid-based nanoparticles

The most established form of nanodelivery agents are lipid-based (Langer and Folkman, 1976; Adams et al., 2017). Lipid-based nanoparticles (LNPs) have been approved by the FDA for delivery of siRNA to the liver and for mRNA vaccine delivery (Paunovska et al., 2022). LNPs are based on structures that can be formed by phospholipids in aqueous solutions, such as micelles and liposomes (Paunovska et al., 2022). Traditional LNPs contain multiple lipids, including cationic lipids, amphipathic phospholipids, and poly (ethylene glycol) (PEG), as well as cholesterol (Semple et al., 2010; Cheng et al., 2015; Paunovska et al., 2022). Variations of these components can be used to direct LNP uptake to specific vascular beds (Paunovska et al., 2018). LNPs can be used to deliver a range of RNA payloads from siRNA to mRNA (Musunuru et al., 2021; Rothgangl et al., 2021).

Because systemic delivery of RNA often leads to accumulation in the liver (Li et al., 2020), various strategies have been developed to target other organs, including the lung. One strategy has been to covalently conjugate an antibody that binds plasmalemma vesicle-associated protein (PV1) to LNPs. This strategy can increase the protein expression of mRNA delivered via these antibody-conjugated LNPs by 40-fold in the lung (Li et al., 2020). Nanoparticle size is an important factor in lung localization, with 160 nm conjugated LNPs leading to greater protein expression in the lungs compared to smaller 70 nm particles (Anselmo et al., 2015; Li et al., 2020). Similarly, the mRNA:lipid ratio is also an important factor in protein expression from delivered mRNA, with a 3% mRNA:lipid ratio showing a ten-fold increase in mRNA expression over a 10% mRNA:lipid ratio (Kranz et al., 2016; Li et al., 2020).

Specific cell types within the lung including epithelial cells, endothelial cells, and immune cells along with cells outside the lung, such as hematopoietic stem cells can be targeted by specific LNP formulations (Paunovska et al., 2022). A strategy of selective organ targeting has been used to systematically generate multiple classes of lipid nanoparticles that can target the lung, liver or spleen and specific cell types such as epithelial cells, endothelial cells, B cells, T cells and hepatocytes (Cheng et al., 2020; Wang et al., 2023). These methodologies allow the engineering of modified lipid nanoparticles to efficiently deliver ribonucleoprotein complexes (RNPs), including CRISPR/Cas9 RNPs, for multiplex editing of genes in mouse lungs (Wei et al., 2020).

In addition to endothelial uptake in the lung (Dahlman et al., 2014; Khan et al., 2018; Cheng et al., 2020), LNPs have been developed for more effective delivery to the lung via nebulization. Delivery of mRNA encoding a neutralizing antibody to haemagglutinin via optimized LNPs could protect mice from a lethal challenge of the H1N1 influenza A virus (Lokugamage et al., 2021). Systematic evaluation of multi-component LNPs has led to the development of LNPs that enable tissue-selective RNA delivery to the lung, even with intravenous administration (Liu et al., 2021). Similar approaches have developed LNPs to deliver mRNA with traditional (Cre recombinase) and CRISPR-Cas9 gene editing tools *in vivo* to lung epithelium via inhalation (Li et al., 2023). Compared to DNA-based viral gene therapy, this strategy avoids long-term expression of genome editors, which can lead to the accumulation of off-target genome mutations. Compared to viral vectors, which elicit an adaptive immune response, this strategy also allows for repeated delivery.

### 3.2 Polymer and polymer-based nanoparticles

A polymer is a macromolecule that consists of multiples of a simpler chemical unit called a monomer and can be natural or synthetic. Polymers are good delivery vehicles because they are biocompatible and have simple formulation parameters that can adopt a variety of possible structures and characteristics (Mitchell et al., 2021). Basic traits such as charge, degradability and molecular weight can be varied to generate desired characteristics (Paunovska et al., 2022). Polymers such as poly (ethylenimine) (PEI), poly (L-lysine) (PLL) and poly (beta-amino-ester) (PBAE) can complex to the anionic phosphodiester backbone of RNA via cationic amine groups. Polymers that do not contain cationic groups can be designed to contain separate cationic groups to serve the same purpose.

The respiratory system presents an accessible but challenging drug delivery target. While there is direct access to the environment through the airways, biological barriers designed for protection against foreign materials, such as mucus, must be overcome for effective delivery. PBAE nanoparticles containing mRNA administered intranasally in mice show expression of the protein product and no evidence of toxicity, such as granuloma or chronic inflammation, for up to 3 months (Su et al., 2011). PBAEs have been used to design stable biodegradable DNA nanoparticles that can penetrate highly adhesive human mucus gel layers and could achieve stable and repeatable transgene delivery in a mouse model (Mastorakos et al., 2015). These PBAE based polyplexes delivered via aerosol can have localized delivery without evidence of reporter mRNA expression in other tissues (Patel et al., 2019).

Other polymer-based nanoparticles can also target the lung. Nanoparticles with multi-modular peptides with three different functional modules and poloxamine polymers are able to deliver co-packaged payloads of mRNA and pDNA for CFTR gene knock-in *in vivo* (Guan et al., 2019). The combination payload consisted of a sleeping beauty transposon containing CFTR sequences and a transposase-encoding mRNA. An advantage of this RNA-based system for gene knock-in is that the transposase is expressed only temporarily and reduces the risk of ongoing insertional mutations (Kebriaei et al., 2017). Importantly, there were fewer integration sites within RefSeq mouse genes compared to lentiviral or adeno-associated viral vectors.

To achieve site-specific editing of CFTR, poly (lactic-co-glycolic) acid (PLGA) nanoparticles have been used to deliver peptide nucleic acids (PNAs) to correct F508del CFTR mutation *in vitro* in human bronchial epithelial cells and *in vivo* in a CF murine model (McNeer et al., 2015). Airway mucus can pose a challenge to drug delivery in cystic fibrosis and other lung diseases. Hybrid lipid-polymer nanoparticles comprising a PLGA core and a dipalmitoylphosphatidylcholine (DPPC) shell engineered for inhalation can penetrate the mucous barrier and release PNA cargo within the cytoplasm (Comegna et al., 2021). Furthermore, polymer-based nanoparticles have been designed that can deliver mRNA to immune cells within the lung. These nanoparticles can be administered systemically and may be helpful for pulmonary-specific immunomodulation (Ke et al., 2020).

### 3.3 Cell-penetrating peptides

Peptide nanoparticles leverage amino acid sequences to impart properties such as structure, charge, solubility, and polarity, which can facilitate interactions with RNA and cellular uptake (Chow et al., 2020). KL4 peptides, which mimic surfactant protein B, can be formulated as a dry powder and deliver both siRNA and mRNA to the lung (Qiu et al., 2017; Qiu et al., 2019). Peptide-based delivery vehicles can also be formulated to target specific cell types. For example, complexes of oligoarginine micelles with high mobility group (HMG) peptide ligands can target activated alveolar macrophages (Choi et al., 2020).

### 3.4 Hybrid delivery systems

Hybrid systems that contain two or more delivery vectors can benefit from the properties of multiple classes of delivery vehicles. These include lipid-polymer hybrids, lipid-peptide hybrids and polymer-peptide hybrids, amongst other possibilities (Chow et al., 2020). Lipid-polymer nanoparticles can facilitate enhanced lung retention for siRNA *in vivo* (Thanki et al., 2019).

Layer-by-layer assembly of hybrid delivery systems allows the development of purpose-built solutions for special applications such as pleural disease. Surface-fill hydrogel nanocomposite is a materials platform developed to deliver microRNA to complex anatomic surfaces locally. SFH requires two polymers prepared in a two-stage process (Majumder et al., 2021). The first stage involves the assembly of a novel peptide-based miRNA nanoparticle whereby an intrinsically disordered cationic peptide is complexed with double-stranded miRNA mimics with a chemically modified anionic backbone. In the second stage, these particles are encapsulated into a shear-thinning, self-assembling hydrogel. This surface-fill hydrogel (SFH) can be sprayed or injected onto large, anatomically complex surfaces such as the pleura to deliver RNA (Choi et al., 2022).

A key feature of SFH is that it can fill small gaps and crevices during application and change its shape after it has been applied. After application, the positively charged peptide-miRNA nanoparticles (∼150 nm diameter) are released from the net-positively charged hydrogel matrix to the adjacent tissues. The design of the peptide-miRNA nanoparticle, including its surface charge state, size, miRNA encapsulation efficiency, and conformation, are important factors in clathrin-mediated cell entry and endosomal trafficking. This platform has been applied in models of malignant pleural mesothelioma (MPM), an aggressive cancer that arises from the mesothelial surface of the pleural cavities (Cho et al., 2021). There is a high rate of recurrence of local disease because MPM covers the pleural surface in a sheet-like manner that technically precludes tumour-free margins, and systemic therapies cannot access sites of residual disease (Baldini et al., 2015). SFH nanocomposites containing either miR-215 or miR-206 mimics reduce rates of local recurrence in pre-clinical models of MPM without detectable miRNA in circulating plasma or observable delivery of miRNA to off-target organs (Singh et al., 2019; 2021; Majumder et al., 2021; Choi et al., 2022).

## 4 Clinical trials in lung disease

With progress and promise shown in pre-clinical studies, RNA-based therapies are now being explored in clinical trials across several domains of lung disease: infectious disease, cancer, and airway disease (Table 1). RNA payloads, including mRNA, miRNA, siRNA and ASO, have been delivered in these trials. Beyond mRNA vaccines for SARS-CoV-2, RNA-based therapies have yet to be approved for lung disease, and there are few ongoing clinical trials in this growing field (Table 2). These trials suggest acceptable tolerability and toxicity profiles across classes of RNA-based therapy, highlight the ability to target specific genes and reflect the biological challenge of treating complex polygenic disease.

**TABLE 1:** Overview of clinical trials investigating RNA therapies in lung disease.

Type of disease	Name	Treatment	Genetic/protein target	Delivery vehicle	Administration method	Disease	Number of patients	ClinicalTrials.gov identifier	Phase
**Infectious Disease**									
	BNT162b2	mRNA	SARS-CoV2 spike glycoprotein	Lipid nanoparticles	Vaccine	COVID-19	43,448	NCT04368728	Approved
	mRNA-1273	mRNA	SARS-CoV2 spike glycoprotein	Lipid nanoparticles	Vaccine	COVID-19	30,000	NCT04470427	Approved
	mRNA-1273.214	mRNA	SARS-CoV2 spike glycoprotein w/Omicron proteins	Lipid nanoparticles	Vaccine	COVID-19	5,158	NCT04927065	Approved
	ALN-RSV01	siRNA	RSV nucleocapsid (N) protein’s mRNA	Lipid nanoparticles	Nasally	RSV	88	NCT00496821	II
	ALN-RSV01	siRNA	RSV nucleocapsid (N) protein’s mRNA	Lipid nanoparticles	Aerosolized	Bronchiolitis obliterans syndrome (BOS) in recipients of lung transplant	87	NCT01065935	IIa
**Cancer**									
	Aprinocarsen	ASO	Inhibits PKC-alpha	-	Orally	NSCLC	14		I
	Aprinocarsen	ASO	Inhibits PKC-alpha	-	Orally	NSCLC	55	NCT00034268	II
	Aprinocarsen	ASO	Inhibits PKC-alpha	-	Orally	NSCLC	880	NCT00034268	III - Terminated
	CV9201	mRNA	Five tumor-associated antigens in non-small cell lung cancer patients (New York esophageal squamous cell carcinoma-1, melanoma antigen family C1/C2, survivin, and trophoblast glycoprotein)	Protamine complexation	Intratumoral Injections	NSCLC	46	NCT00923312	II/IIa
	G3139	ASO	Bcl-2	-	Intravenously	SCLC	16	NCT00005032	I/II
	Atu027	siRNA	PKN3	siRNA lipolex	Intravenously	Advanced solid tumors	27	NCT00938574	I
	TargomiRs	miRNA	EGFR	Minicells	Intravenously	Malignant Pleural Mesothelioma	26	NCT02369198	I
**Airways Disease**									
	QR-010	ASO	CFTR	-	Nasally	Cystic Fibrosis	18	NCT02564354	I
	QR-011	ASO	CFTR	-	Nasally	Cystic Fibrosis	70	NCT02532764	Ib
	MRT5005	mRNA	CFTR	Lipid nanoparticles	Nasally	Cystic Fibrosis	40	NCT03375047	I/II
	TPI ASM8	ASO	CCR3 & βc	-	Nasally	Asthma	16	NCT01158898	II
	AIR645	ASO	IL-4/IL-13 Receptor (α chain)	-	Nebulization	Asthma	80	NCT00658749	I

**TABLE 2:** Overview of ongoing clinical trials investigating RNA therapies in lung disease.

Type of study	Name	Treatment	Genetic/protein target	Delivery vehicle	Administration method	Disease	Number of patients	ClinicalTrials.gov identifier	Phase
**Active & Recruiting**									
	VX-522	mRNA	CFTR	Lipid nanoparticle	Oral inhalation with nebulizer	Cystic Fibrosis	Recruiting	NCT05668741	I
	ARCT-032	mRNA	CFTR	Lipid nanoparticle	Oral inhalation with nebulizer	Cystic Fibrosis	Recruiting	NCT05712538	I
	mRNA personalized tumor vaccine	mRNA	Neoantigens	-	Vaccine	NSCLC	Recruiting	NCT03908671	-
**Active**									
	RSVpreF + BNTb162b2	mRNA	SARS-CoV2 spike glycoprotein and RSV prefusion-F protein	Lipid nanoparticle	Vaccine	COVID-19 and RSV	1,079	NCT05886777	II
	mRNA-1345	mRNA	Stabilized prefusion F glycoprotein	Lipid nanoparticle	Vaccine	RSV	36,557	NCT05127434	II/III
	CL-0059 or CL-0137	mRNA	Stabilized prefusion F glycoprotein	Lipid nanoparticle	Vaccine	RSV	790	NCT05639894	I/II
	mRNA-1345	mRNA	Stabilized prefusion F glycoprotein	Lipid nanoparticle	Vaccine	RSV	651	NCT04528719	I
	mRNA-1045 or mRNA-1230	mRNA	Influenza virus proteins, SARS-CoV2 spike glycoprotein and RSV prefusion-F protein	Lipid nanoparticle	Vaccine	Influenza, RSV, SARS-CoV-2	392	NCT05585632	I

### 4.1 Infectious disease

The most successful application of RNA-based therapy in lung disease has been the adoption of mRNA vaccines for SARS-CoV-2 and the COVID-19 pandemic (Hall et al., 2021; Zhang et al., 2022). mRNA vaccines were one of three major vaccine platforms for SARS-CoV-2, alongside vaccines based on viral vector and recombinant protein plus adjuvant (Pollard and Bijker, 2021). Two mRNA vaccines, BNT162b2 (Pfizer) and mRNA-1273 (Moderna) were rapidly developed (Walsh et al., 2020) and demonstrated remarkable effectiveness, with early vaccine efficacy of 95% for BNT162b2 (Thomas et al., 2021) and 94% for mRNA-1273 (Baden et al., 2021). However, these numbers decline to 67% and 75%, respectively, at 5–7 months (Rosenberg et al., 2022). The pivotal trial that contributed to emergency FDA approval of BNT162b2 enrolled 43,448 participants aged 16 and older, along with 2,264 participants aged 12–15 (Polack et al., 2020). Both vaccines are encapsulated in lipid nanoparticles, code for the spike glycoprotein (Baden et al., 2021), and elicit CD4^+^ and CD8^+^ T-cell responses (Jackson et al., 2020; Zhang et al., 2022). The common side effects observed were injection site pain, headaches, fatigue, fever, chills, muscle pain, nausea, and vomiting (Baden et al., 2021).

Other anti-infective strategies aimed at treating infections have also been explored. ALN-RSV01 is an siRNA directed against the nucleocapsid (N) mRNA of the respiratory syncytial virus (RSV). A proof-of-concept randomized, double-blind, placebo-controlled trial compared saline nasal spray with a nasal spray of ALN-RSV01 2 days before and 3 days after experimental RSV infection in 88 healthy subjects and showed a 38% decrease in culture-defined RSV infections. ALN-RSV01 had a similar safety profile to placebo, and the antiviral effect was independent of pre-existing antibodies and intranasal cytokines (DeVincenzo et al., 2010). RSV infection is associated with an increased incidence of bronchiolitis obliterans syndrome (BOS) in lung transplant recipients. ALN-RSV01 has also been evaluated in a phase IIb randomized, double-blind, placebo-controlled trial of 87 lung transplant recipients. There was a trend toward a decrease in new or progressive BOS in the modified intention-to-treat analysis, which was significant in the per-protocol cohort (Gottlieb J et al., 2016). These results show potential for siRNA-therapeutics in the treatment of lung infection.

### 4.2 Cancer

In thoracic cancers, a range of RNA-based approaches, from antisense therapies to cancer vaccines, have been assessed in clinical trials. Early reports of RNA-based therapy in lung malignancy focused on ASOs (Coudert et al., 2001). In an early study of the anti-c-raf ASO, ISIS-5132, as monotherapy, toxicity was considered acceptable, but there was no clinical benefit seen in 18 patients with non-small cell lung cancer (NSCLC) (Coudert et al., 2001). A follow-up phase I trial showed that a combination of paclitaxel, carboplatin, and ISIS-5132 was well tolerated but showed no objective responses in 13 patients (Fidias et al., 2009). Subsequently, custirsen, an ASO targeting the gene clusterin (CLU), showed promise in Stage IIIB/IV non-small cell lung cancer (NSCLC) in a Phase I clinical trial for use in combination with cisplatin and gemcitabine (Laskin et al., 2012). Several trials have evaluated aprinocarsen, an ASO directed against protein kinase C-alpha, in combination with chemotherapy in advanced NSCLC (Vansteenkiste et al., 2005; Paz-Ares et al., 2006; Ritch et al., 2006). In a large Phase III trial of 670 patients that was stopped early due to results of another trial, aprinocarsen did not enhance survival compared to gemcitabine and cisplatin with advanced NSCLC (Paz-Ares et al., 2006). Despite early challenges, other ASOs have been evaluated over a decade later. Apatorsen (OGX-427) is an ASO directed against heat shock protein 27 (Hsp27) mRNA and was evaluated in a randomized, double-blinded, Phase II trial of 155 patients in combination with carboplatin and pemetrexed. Apartorsen was well tolerated but did not improve outcomes in patients with metastatic nonsquamous NSCLC as first-line treatment (Spigel et al., 2019).

Other groups have assessed ASOs in small cell lung cancer (SCLC). G3139 (oblimersen) is an ASO directed against Bcl-2 and underwent a pilot clinical trial (Rudin et al., 2002), and a subsequent Phase I trial, both of which showed tolerability and potential for treatment response (Rudin et al., 2004). Despite this early success, a Phase II trial of oblimersen in combination with carboplatin and etoposide showed worse outcomes in the group with oblimersen, despite evidence supporting a critical role for Bcl-2 in treatment resistance SCLC (Rudin et al., 2008). Inadequate suppression of Bcl-2 *in vivo* was proposed as a reason for the lack of efficacy.

The first clinical experience for miRNA-based therapy in thoracic malignancy was the assessment of mIR-16-based miRNA mimics in malignant pleural mesothelioma (MPM). A Phase I dose escalation open-label trial assessed TargomiRs, a term describing proprietary miRNA mimics based on the miR-16 seed sequence that are encapsulated in a bacterial-derived mini-cell vector targeted to the epidermal growth factor receptor (EGFR) by panitumumab-based antibodies. Of 22 patients who were assessed for response, 1 achieved an objective response to therapy by CT (van Zandwijk N et al., 2017).

Cancer immunotherapy is designed to elicit an immune response to tumour antigens. In stage IIIB/IV NSCLC, a phase I/IIa trial assessed the safety of CV9201, an mRNA-based immunotherapy encoding five NSCLC tumour antigens (New York esophageal squamous cell carcinoma-1, melanoma antigen family C1/C2, survivin, and trophoblast glycoprotein) (Sebastian M et al., 2019). This application highlights the potential for multiplexing drug delivery for RNA-based therapies. Promise for this therapeutic avenue was shown as no serious adverse events occurred, and immune responses were detected after treatment.

### 4.3 Airways disease

Most approved RNA-based therapies target monogenic disorders (Egli and Manoharan, 2023). In the lung, cystic fibrosis (CF) represents an attractive target for the design of RNA-based therapeutics, with mutations of the cystic fibrosis transmembrane receptor (CFTR) being the causative factor in the development of the disease (Riordan et al., 1989; Ong and Ramsey, 2023). Eluforsen (QR-010) is an ASO designed to bind CFTR mRNA around the ΔF508 mutation and lead to the translation of wild-type CFTR protein (Sermet-Gaudelus et al., 2019). An open-label study of intranasal eluforsen three times weekly for 4 weeks showed improved chloride transport in a ΔF508 homozygous cohort (Sermet-Gaudelus et al., 2019) and was followed by a randomized, double-blind, placebo-controlled dose escalation Phase Ib trial of adult CF found that eluforsen was well tolerated, with low systemic exposure and improvement in the Cystic Fibrosis Questionnaire-Revised Respiratory Symptom Score after 4 weeks (Drevinek et al., 2020).

In theory, mRNA coding for CFTR could be helpful with any CFTR mutation. MRT5005, a codon-optimized CFTR mRNA delivered by inhaled lipid nanoparticles, is being evaluated in a randomized, double-blind, placebo-controlled Phase I/II study. Interim analysis of 42 subjects found 14 febrile reactions over 28 days, most of which resolved within 1–2 days and allowed ongoing treatment. FEV1 remained stable, but no beneficial effects on FEV1 were observed (Rowe et al., 2023).

ASOs have also been used to target specific factors contributing to airways inflammation in asthma (Gauvreau et al., 2011). Early development of an A1 adenosine receptor ASO, EPI-2010, showed modest efficacy in mild asthma, but product development was discontinued without additional benefit in a Phase II trial (Ball et al., 2003; Liao et al., 2017). TPI-ASM8 contains two ASOs that, together, target the beta subunit of IL-3, IL-5 and GM-CSF receptors and the chemokine receptor CCR3. In a single-center, open-label, ascending dose study, inhaled TPI-ASM9 was well tolerated in stable asthma, and decreased airway responsiveness to methacholine challenge and decreased sputum eosinophils in response to inhalational challenge (Gauvreau et al., 2008; Gauvreau et al., 2011).

## 5 Future directions

The evolution of chemistry for RNA-based therapeutic payloads and for nanotechnology-based delivery vehicles have advanced the applicability of RNA-based treatments to human disease. By leveraging the ability to selectively target different compartments within the lungs (e.g., airways, alveoli, pleural cavity) and specific cell types (e.g., epithelium, endothelium, immune cell), RNA therapy offers enhanced precision in targeting lung disease (Paunovska et al., 2022). Universal delivery platforms could accelerate the translation of biological findings (Chow et al., 2020), whereas made-for-application platforms could provide the most effective means of drug delivery and targeting. The ability to rapidly develop new therapeutics based on nucleotide sequence reduces the duration of drug development, such that deconvoluting disease biology poses a greater challenge than druggability.

### 5.1 Airways disease

In obstructive airway diseases, such as asthma and chronic obstructive pulmonary disease (COPD), treatment delivered by inhalation can enhance local delivery while decreasing systemic exposure (Chow et al., 2020). Naked RNA can transfect lung epithelium, though delivery vehicles that enhance transfection efficiency are still a preferred route (Chow et al., 2020). Disease-specific formulations may be important considerations as fluid and pulmonary surfactants can influence drug delivery (Hidalgo et al., 2015), and assessment of fluids may become a feature of individualized therapy. Through efficient delivery methods, it becomes possible to modulate gene expression, reduce inflammation, and improve lung function in patients with airway diseases with absent or limited systemic administration (Patel et al., 2019). Activated T-helper cells subtype 2 (TH2), which contribute to asthma, have been targeted by conjugated polymer nanoparticles in a dry powder formulation. Anti-GATA3 siRNA can be delivered via this system to reduce the production of multiple cytokines en masse rather than blocking or downregulating single cytokines (Keil et al., 2020). As single-cell technologies improve the resolution of disease pathogenesis (Adams et al., 2023; Lim et al., 2023), these targeted approaches will provide therapeutic options that more closely address pathologic processes and cell types while reducing side effects.

### 5.2 Lung transplantation

Lung transplant recipients face the challenge of maintaining graft function and preventing rejection. Specific intragraft immune cell populations are of prognostic and likely mechanistic importance in the development of chronic lung allograft dysfunction (Bos et al., 2022; Beber et al., 2023). Here, as with asthma, RNA therapy using specific delivery vehicles can play a crucial role by specifically targeting immune cells in the lungs (Ke et al., 2020). Through the development of precise delivery methods that home in on these immune cells, it becomes possible to modulate local immune responses and enhance long-term transplant success rates.

### 5.3 Pulmonary vascular disease

Local delivery of RNA-based therapy to the lung via inhalation or to the pleural space are attractive options, yet RNA delivered via these routes may not effectively reach the pulmonary endothelium to benefit pulmonary vascular disorders. However, delivery vehicles have been engineered to target lung endothelium, even with systemic delivery (Dahlman et al., 2014; Khan et al., 2018; Cheng et al., 2020). Loss-of-function mutations in pulmonary arterial hypertension (PAH), such as BMPR2 (Cuthbertson et al., 2023), could be candidates for mRNA-based therapies.

### 5.4 Critical care

Additionally, critical care conditions like acute respiratory distress syndrome (ARDS) require comprehensive treatment approaches that address both local lung inflammation and systemic consequences. RNA therapy delivered systemically holds promise in treating ARDS (Allegra et al., 2023) by targeting inflammatory pathways locally within the lungs and systemically. In single-cell RNA sequencing of severe COVID-19, loss of a long noncoding RNA (lncRNA) PIRAT in blood monocytes leads to increased inflammatory response by alarmins at the expense of anti-viral response via the JAK-STAT pathway (Aznaourova et al., 2022). These high-resolution insights, facilitated by single-cell technologies, suggest settings which require precise therapeutic targeting that is possible with RNA-based approaches.

### 5.5 Interstitial lung disease

Recently, the treatment of interstitial lung disease (ILD) has been driven by the recognition of progressive fibrosis as a feature of several ILDs. However, multiple clinical, radiographic, and histologic patterns exist, reflecting distinct pathologic processes (Renzoni et al., 2021). Multiple contributing cell types and gene targets, including miRNAs, are being identified and could lead to a molecular classification of ILD entities (Renzoni et al., 2021). These insights suggest potential benefits for an array of RNA payloads and delivery vehicles to target relevant pathogenic processes and alter functional trajectory in ILD.

### 5.6 Long noncoding RNAs as targets for RNA-based therapy in the lung

Noncoding RNAs have emerged as major products of the human genome and are involved in all cellular functions. Many classes of noncoding RNA exist, many of which have specific functions and mechanisms, such as miRNAs. Long noncoding RNAs (lncRNAs) have traditionally been defined as noncoding transcripts of more than 200 nucleotides, though a new consensus statement proposes that they be defined as >500 nt to better distinguish from other noncoding RNAs (Mattick et al., 2023). Notably, lncRNAs are likely the major output of genomes in complex organisms and annotated human lncRNAs in the GENCODE database number 19,928 - a number greater than the number of annotated protein-coding genes (GENCODE, 2022). LncRNAs are attractive therapeutic targets as they generally have more specific expression patterns than protein-coding genes (Derrien et al., 2012).

Emerging evidence is defining roles for lncRNAs in lung development, homeostasis and disease. For example, the lncRNA LL18/NANCI contributes to saccule formation/alveolarization in mouse lungs (Herriges and Morrisey, 2014). In hypoxia, the lncRNA GATA2-AS1 was shown to modulate HIF-1𝛂 and HIF-2𝛂 balance and hypoxia signalling in vascular endothelium (Man et al., 2023). The lncRNA TYKRIL is overexpressed in hypoxia and idiopathic pulmonary arterial hypertension (iPAH) and contributes to a pro-proliferative and anti-apoptotic phenotype in pulmonary artery smooth muscle cells and pericytes (Zehendner et al., 2020). Similarly, the lncRNA SMILR also contributes to smooth muscle proliferation in hypoxic conditions and can be detected in the serum of PAH patients (Lei et al., 2020). These examples illustrate the potential for lncRNAs as specific and effective targets of therapy in lung disease through RNA-based therapies.

## 6 Conclusion

In conclusion, the future of RNA therapy in lung diseases is promising and offers new avenues for effective treatment. The ability to target specific compartments within the lungs, in combination with various RNA payloads, provides versatile options for precise and personalized therapy. We have briefly overviewed various RNA payloads, delivery vehicles and the clinical state of RNA-based therapy in the lung. Few clinical trials have been completed, and vaccines directed at SARS-CoV-2 are the only FDA-approved treatments. Thus, continued research and development in this field are crucial to fully realize the transformative potential of RNA therapy in the management of lung diseases.
